# How Learning Styles Characterize Medical Students, Surgical Residents, Medical Staff, and General Surgery Teachers While Learning Surgery: Scoping Review

**DOI:** 10.2196/66766

**Published:** 2025-09-05

**Authors:** Gabriela Gouvea Silva, Marco Antonio Ribeiro Filho, Carlos Dario da Silva Costa, Stela Regina Pedroso Vilela Torres de Carvalho, Joao Daniel de Souza Menezes, Matheus Querino da Silva, William Donega Martinez, Bruno Cardoso Goncalves, Natália Almeida de Arnaldo Silva Rodriguez Castro, Luiz Vianney Cidrão Nunes, Emerson Roberto Santos, Helena Landim Gonçalves Cristóvão, Alexandre Lins Werneck, Alex Bertolazzo Quitério, Sonia Maria Maciel Lopes, Denise Vaz-Oliani, Fernando Facio, Patrícia da Silva Fucuta, Alba Regina de Abreu Lima, Vania M S Brienze, Heloisa Cristina Caldas, Julio Cesar Andre

**Affiliations:** 1 Center for Studies and Development of Health, Faculdade de Medicina de São José do Rio Preto, Avenida Brigadeiro Faria Lima, 5416, São José do Rio Preto, 15090-000, Brazil, 55 17982022252; 2 Reproductive Medicine Center of University Hospital Center Cova da Beira, University of Beira Interior, Covilhã, Portugal; 3 Epidemiology Department, Faculdades Integradas de Ciências, Educação, Cultura e Administração de Ceres (FACERES), São José do Rio Preto, Brazil

**Keywords:** learning styles, general surgery, medical saff, surgical residents, students, medical faculty, medical school, cognitive theories, scoping review, PRISMA

## Abstract

**Background:**

Learning style is a biologically and developmentally imposed configuration of personal characteristics that makes the same teaching method effective for some and ineffective for others. Studies support a relationship between learning style and career choice, resulting in learning style patterns observed in distinct types of residency programs, which can also be applied to general surgery, from medical school to the latest stages of training. The methodologies, populations, and contexts of the few studies pertinent to the matter are very different from one another, and a scoping review on this theme will unequivocally enhance and organize what is already known.

**Objective:**

The goal of this study is to identify and map out data from studies that report on learning styles in medical students, surgical residents, medical staff, and surgical teachers.

**Methods:**

The search strategy was performed on September 25, 2023, by a librarian and digital search strategy expert, through the descriptors “learning, style” and “surgery.” The databases consulted were Embase, SCOPUS, Web of Science, and PubMed through descriptors and their synonyms, according to MeSH (Medical Subject Headings). Of the 213 articles found, 135 articles remained after the exclusion of duplicates. The remaining 78 articles were analyzed by 3 of the researchers independently. A total of 27 articles were selected, and 2 articles were excluded because the full article was not found.

**Results:**

A total of 25 articles were included in the review. A total of 96% (n=24) of the articles used cognitive theories as their theoretical basis. Regarding learning style instruments, 36% (n=9) articles used the visual, aural, read, and kinesthetic learning method instrument, and 40% (n=10) articles chose Kolb’s learning style inventory. The papers concentrate especially on the 2010s, and most of them are from North America (16/25, 64%) or Europe (6/25, 24%). The smallest study had 15 participants and the biggest had 1549 participants. The included studies primarily focused on surgical residents (21/25, 84%), with fewer targeting faculty and staff (9/25, 36%). The primary objectives of the studies were to investigate the relationship between learning styles and performance (15/25, 60%), gender differences (7/25, 28%), changes over time (4/25, 16%), and motivation (3/25, 12%).

**Conclusions:**

This scoping review reveals a limited and geographically concentrated body of research on learning styles in surgery education, primarily focusing on surgical residents and using Kolb’s learning style inventory and visual, aural, read, and kinesthetic learning method instruments. Considerable gaps exist regarding geographical diversity and the study of medical staff and faculty. These findings underscore the need for future research with a broader scope to better inform educational strategies in surgery.

## Introduction

The concept of learning styles was first developed as a result of the interest in individual differences through the learning process, at the beginning of the 1960s [1]. According to Dunn, everyone has a peculiar learning style, like a signature. In this regard, tailoring teaching to different learning styles may help and improve results in education.

Several theories and inventories were created to assess and determine one’s learning style, including the following: Kolb’s model, Felder and Silverman’s model, and Gregorc’s model, especially in health care education. A debate about whether learning styles are fixed or flexible exists to this day, and to what extent the context can influence and determine them [2].

However, it is important to acknowledge the growing body of evidence that challenges the effectiveness and validity of learning styles theories. While these theories suggest that tailoring instruction to individual learning preferences can enhance learning outcomes, empirical studies have shown limited support for this approach [3]. Specifically, research indicates that many students do not study in ways that align with their self-reported learning styles, and that matching instruction to these styles does not necessarily improve performance [4].

For example, David Kolb describes learning as a process where knowledge is transformed through experience, and that acknowledgment is the combination of appropriation and transformation of experience. His theory is a holistic model called “experiential learning” and emphasizes experience in its core, differing from other theories [5]. Kolb’s scheme hypothesizes that the learner has a concrete experience, upon which he reflects. Through reflection, it is possible to formulate abstract concepts and make appropriate generalizations, then consolidate the understanding by testing the implications of the knowledge in new situations. This then provides a concrete experience, and the cycle continues. Learners with different learning preferences will have different strengths and weaknesses in the quadrants of the (Kolb) cycle [6]. Based on that, he created the learning style inventory (LSI) to determine and assess individually the different learning styles, divided into converging, diverging, assimilating, and accommodating [7].

In medical education, it is particularly important to remember the heterogeneity of students. Some programs count on learners who have already completed a university degree; in others, the students come straight from secondary school, and many face a mixture of both. The broader concept of medical education includes postgraduate students and continuing professional development, too. Each of them will have variable individual constraints, experiences, and preferences [8].

The diversity in educational backgrounds, cultural influences, ethnic origins, and gender identities among contemporary surgical trainees presents unique challenges and opportunities for educators. While it is often assumed that these diverse backgrounds necessitate a personalized approach to learning, it is crucial to recognize that individual learning preferences may not always align with optimal learning strategies [9].

Instead, effective surgical education should focus on evidence-based instructional methods that cater to a wide range of learners, while also promoting critical thinking, adaptability, and lifelong learning skills.

Perry [10] noted that students change their learning approach as they progress through their college years. Students often begin with a “duality” approach, with a clear view between right and wrong, towards “multiplicity,” where they recognize that context is important, and that there are various valuable sources of knowledge and experience.

Knowledge is the main domain of medical education, but the outcome depends strongly on other domains such as attitude, lifelong learning, empathy, communication, ethics, and professionalism. The clinical environment is challenging for both the student and the teacher, without mentioning the patient, who is at the center of the action. In this bigger context, it is vital to use different learning theories to promote effective learning [8].

Contemporary surgical trainees come from diverse educational, cultural, ethnic, and gender backgrounds [11], and are pressured to develop skills not only in the role as a medical expert, but also as a professional, scholar, health advocate, manager, collaborator, and communicator [12].

Educating surgeons is an ancient tradition that has existed since the development of surgery [13], and for centuries, surgical residency curricula have been guided primarily by tradition. The apprenticeship model has been one of the essential components of surgical training. It generally involves 3 steps: assisting at operations, performing operations with expert assistance, and operating without assistance.

Modern surgical education has been revolutionized by exponents such as Halsted: the historical model of apprenticeship was transformed into the current organized system that we call Residency [11].

The present day, however, requires the realization of more complex procedures, performed more regularly and in safer manners, demanding even more prepared professionals [14].

Studies report on a relationship between learning style and medical career choice, resulting in learning style patterns observed in distinct types of residency programs, which can also be applied to general surgery. Based on Kolb’s LSI, students classified as accommodating and diverging frequently chose surgery as their career choice, whereas converging chose internal medicine, and assimilating chose academic medicine [15].

However, despite the theme’s relevance, a preliminary search in the MEDLINE, Cochrane Database of Systematic Reviews, and Joanna Briggs Institute’s evidence synthesis revealed no scoping review. Moreover, the methodologies, populations, and contexts of the few studies pertinent to the matter are very different from one another, and a scoping review on this theme would unequivocally enhance and organize what is already known.

Therefore, the aim of this scoping review is to identify, map, and synthesize the existing literature on learning styles in medical students, surgical residents, medical staff, and surgical teachers within the field of surgery. This review seeks to provide a comprehensive overview of the current state of knowledge, identify gaps in the literature, and inform future research and educational practices in surgical training.

## Methods

### Overview

The scoping review proposed here was carried out according to Arksey and O’Malley’s [16] structure, using the first five stages: (1) identify the research question; (2) identify relevant studies; (3) select studies; (4) map out the data; and (5) collate, summarize, and report the results. Since this is preliminary research, it is likely that more studies on the theme will be finalized. Although the sixth stage of Arksey and O’Malley’s [16] structure (consulting) will not be completed in this review, its results can inform this stage in a future study. The structure is congruent with The Joanna Briggs Institute’s scoping review methodology. The protocol for this scoping review was duly developed in accordance with relevant reporting guidelines and has been publicly registered [17].

### Inclusion Criteria

After a discussion involving the researchers, the eligibility criteria were defined.

#### Participants

Studies were made with medical students, surgical residents, medical staff in general surgery, and the general surgery’s medical faculty. It was not obligatory to contain all the population’s extracts. Studies were included if they involved at least one of these population groups; it was not required for a study to include all listed groups.

#### Concept

The included studies approached “learning styles” of the target population, regardless of the chosen instrument to define them.

#### Context

The eligible studies were those related to teaching surgery to the population in question, in any country.

### Types of Sources

The following types of sources were included in the review: studies with qualitative and quantitative approaches, primary studies, systematic reviews, meta-analyses and meta-synthesis, books, and guidelines, published in indexed sources.

### Research Strategy

The search strategy was performed on September 25, 2023, by a librarian and digital search strategy expert, through the descriptors “learning, style” and “surgery.” There was no time frame restriction in the search. For the combination of descriptors, the Boolean operators “AND” and “OR” were considered. Words were reduced to their root to embrace variations in writing and broaden the search scope.

Databases consulted were Embase, SCOPUS, Web of Science, and PubMed through descriptors and their synonyms—according to the Medical Subject Headings—to every strategy item. These databases were selected because they are comprehensive and have a broad coverage of health publications. The databases’ search resulted in a table made using a Microsoft Excel spreadsheet. In accordance with PRISMA-P (Preferred Reporting Items for Systematic Reviews and Meta-Analyses Protocols) guidelines, the detailed search strategy used for one database (eg, PubMed) is provided in Multimedia Appendices 1 and 2 including specific search terms, Boolean operators, and any applied limits, to ensure reproducibility.

### Data Selection and Extraction

The articles were evaluated by 3 independent researchers after discussions about inclusion and exclusion criteria. To align the eligibility criteria among the researchers, the titles and abstracts of 25 random articles were analyzed by 3 of the researchers. Disagreements regarding the inclusion or exclusion of the articles were discussed until a consensus was reached. There was a 100% agreement concerning the inclusion and exclusion of the articles. After the articles’ selection, a form was used to extract data from the articles’ full analysis, both quantitatively and qualitatively.

After the articles’ selection, a form was used to extract data from the articles’ full analysis, both quantitatively and qualitatively.

## Results

### Overview

**Figure 1.**
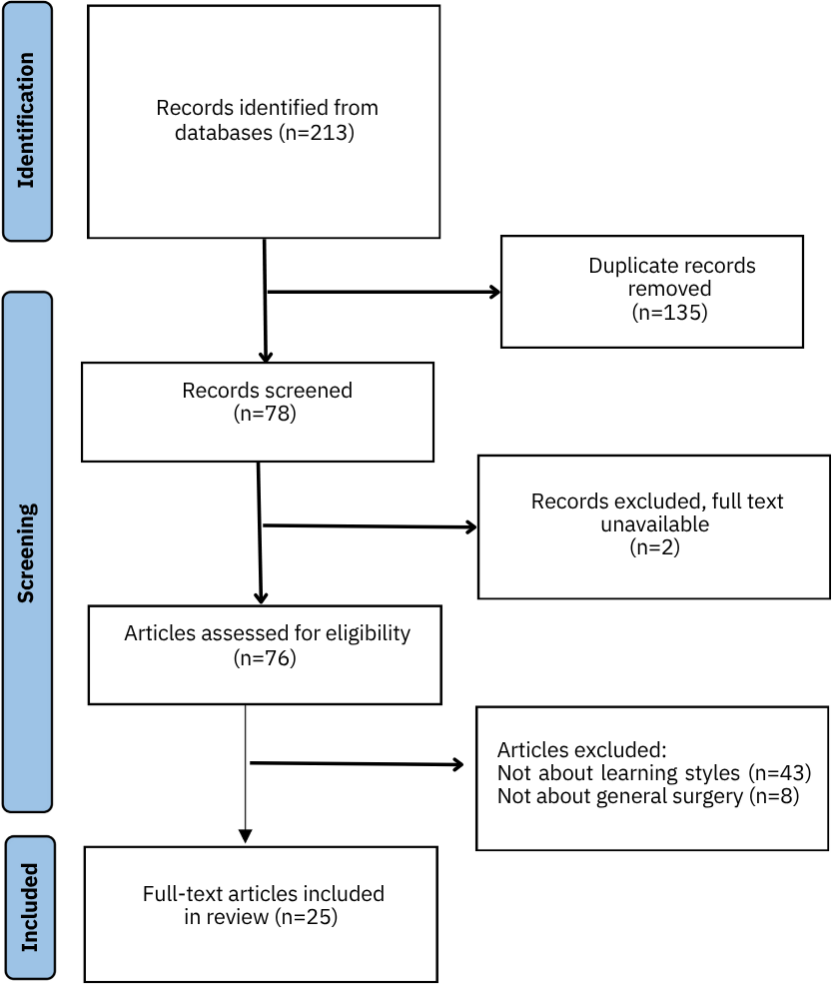
PRISMA-ScR (Preferred Reporting Items for Systematic Reviews and Meta-Analyses extension for Scoping Reviews) flow diagram of learning styles in general surgery.

Of the 213 articles found, 135 remained after the exclusion of duplicates. The remaining 78 articles were analyzed by 3 of the researchers independently, from which 27 articles were selected, and 2 articles were excluded because the full article was not found. A PRISMA-ScR (Preferred Reporting Items for Systematic Reviews and Meta-Analyses extension for Scoping Reviews; Checklist 1) flow diagram was produced (Figure 1). Thus, the analysis included a total of 25 articles.

### Learning Theory and Learning Style Instrument

Regarding the theoretical basis, 96% (n=24) of the included articles used cognitive theories as a theoretical basis. Regarding the choice of learning style instrument: 36% (n=9) used the visual, aural, read, and kinesthetic learning method (VARK) instrument, and 40% (n=10) chose Kolb’s LSI. One study opted for the Multiple Intelligences Developmental Assessment Scales [18] instrument; another used the Meyer-Briggs type indicator [19]. One study used the students’ learning preferences questionnaire [20], and 2 others chose the 24 self-reported LSI questionnaire [21,22]. One study was conducted as a review, so it explained various learning styles approaches [23].

### Mapping Studies Around the World

**Figure 2.**
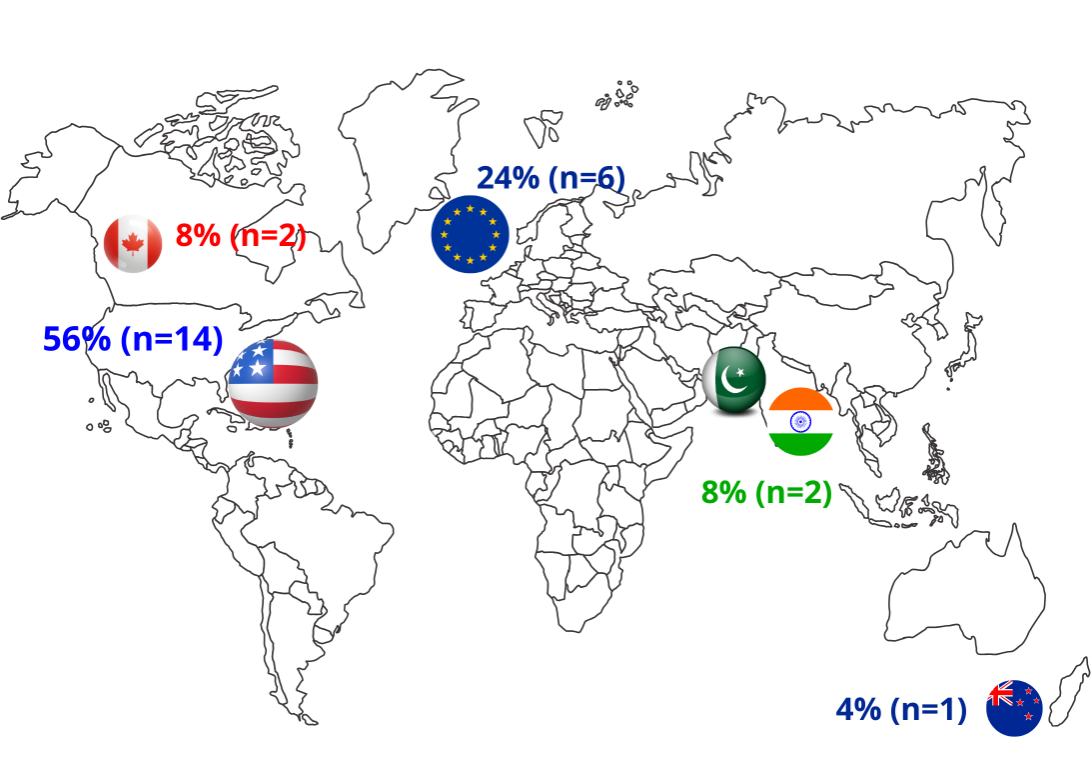
Map of previous studies conducted around the globe regarding learning styles and surgery.

A majority of the studies were conducted in North America or Europe. Specifically, 14 studies were developed in the United States and 2 studies were from Canada, accounting for 64% (n=16) of the studies with a northern American population. A total of 24% (n=6) of the studies took place in Europe, 4 studies in the United Kingdom, one study in Spain [24], and one study in the Netherlands [23]. Only one study was conducted in Oceania (New Zealand) [18], and the other 2 studies were from Asia (2/25, 8%), one from Pakistan [20] and the other from India [21]. No studies (0/25, 0%) were found regarding learning styles and surgery in Latin America or Africa. Figure 2 summarizes these findings.

### Interest in Learning Styles Across Time

Learning styles were not a common subject until the 1960s. Table 1 shows the number of articles on the theme in each decade. The year 2010 was the most interesting in the subject, with 15 published papers.

**Table 1.** Number of articles about learning styles in surgery through the decades.

Year	Articles, n
1970	1
2000	4
2010	15
2020	4

### Characteristics

Most articles had a transversal design, accounting for 88% (n=22) of the total. One study (1/25, 4%) was a review, and 3 others (3/25, 12%) were cohorts. The number of participants was, on average, 114. The smallest study had 15 participants [25] and the biggest, 1549 participants [19].

**Figure 3.**
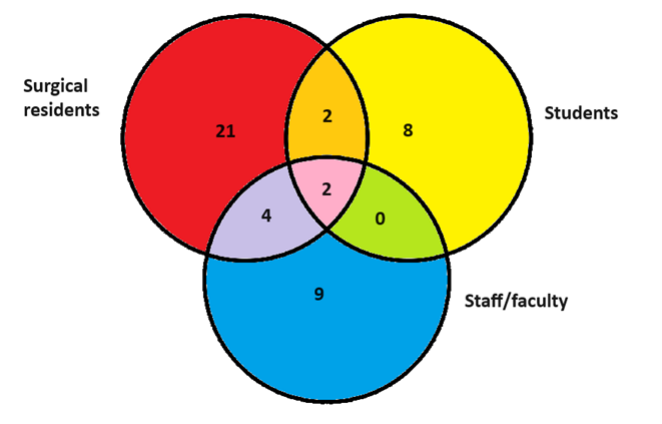
Number of articles with each population.

The articles included students, surgical residents, and surgical staff/faculty. Figure 3 shows the number of articles with each of these populations. Specifically, 84% (n=21) of the papers included general surgery residents, while 36% (n=9) targeted faculty and staff.

The goals of these studies differed a bit. A total of 60% (n=15) of participants were interested in the relation between learning styles and performance, such as laparoscopic or robotic skills, academic achievement, and so on. Another 28% (n=7) of participants also looked over gender gaps and differences among female and male participants. Four studies (4/25, 16%) took an interest in changes in learning styles over time. Three studies (3/25, 12%) also evaluated motivation.

## Discussion

### Principal Findings

This scoping review identified 25 studies published between the 1960s and 2023 that explored learning styles in the context of surgery education. The majority of these studies were published in the 2010s and were predominantly conducted in North America and Europe. The most frequently used instruments to assess learning styles were Kolb’s LSI and the VARK questionnaire. The included studies primarily focused on surgical residents, investigating the relationship between learning styles and various outcomes such as performance, gender differences, changes over time, and motivation.

There are several ways to face adult education. For example, there are multiple theories that can be superposed along with multiple ways of evaluating them according to the chosen theory. In health care, there are several specificities that make the educational process more complex, since there are a lot of abilities and competencies to be developed in a short period of time. On top of it, medical science evolution becomes faster every day, adding challenges to education and the preparation of professionals.

Surgery is commonly known as a delicate place for developing skills and training in high-risk procedures. When teaching surgery, it is important to account for medical students, residents, and continuous improvement for teachers and medical staff. These pieces of surgical educational chess are very different from each other, each one with special needs and goals. To understand how these population extracts capture and keep knowledge is vital to effective learning [26].

As highlighted in the results, Kolb’s LSI and the VARK instrument were the most commonly used tools in the reviewed literature. Kolb’s LSI, based on experiential learning theory, categorizes learners into 4 styles (diverging, assimilating, converging, and accommodating) based on their preferences for concrete experience, reflective observation, abstract conceptualization, and active experimentation [4,5]. The VARK questionnaire, on the other hand, focuses on sensory preferences for information intake and output, classifying learners as visual, auditory, read/write, or kinesthetic [16]. While both instruments aim to identify individual learning preferences, they stem from different theoretical frameworks and assess distinct aspects of the learning process. Kolb’s model is more focused on how individuals process information through a cycle of experience and reflection, whereas VARK is centered on the modality through which information is best received and conveyed. The prevalence of these instruments in the literature may be attributed to their relative ease of administration and interpretation. However, it is important to note that the psychometric properties and theoretical underpinnings of some learning style instruments, including Kolb’s LSI, have faced criticism in the broader educational psychology literature, raising questions about their validity and the implications of findings based solely on these tools [27-29]. Future research should consider these limitations and potentially explore alternative or complementary approaches to understanding individual differences in surgical learning.

The study field of learning styles and surgery is wide and still poorly explored. It is only possible to imagine the benefits that would emerge with the amplification of this understanding in teaching and learning surgery in the next decades.

### Principal Results

Historically, there are not many studies on learning style in surgery, as pointed out by this scoping review. The papers concentrate especially on the year 2010, and most of them are in North America or Europe. Thanks to important cultural, financial, and political contexts, it is evident that more information on the participants from the rest of the world is needed.

The main instruments used in the papers were Kolb’s LSI and the VARK instrument, both derived from cognitive theories. The ease of handling the instruments was probably an impact factor in their choice, on top of their proven effectiveness and reproducibility.

A total of 84% (n=21) of papers had general surgery residents as the target, while only 36% (n=9) targeted faculty and staff. The improvement of teaching goes by deep knowledge of teachers and their methods [30]. So, prospective surgeons should not be the only goal of surgical educational research. The ratio is to disarticulate the former master-learner vision, understanding that everyone involved in the learning process is a potential and continuous learner [16,24].

The main purpose of the papers was to relate learning styles and performance, motivation, gender, and changes over time.

The articles aiming for performance did it in a lot of diverse ways. One way to assess performance is to evaluate the scoring in admission tests, and research has concluded, many times, that a nonspecific learning style is being favored in those types of admissions [25-29]. Others took interest in psychomotor skills, measured by specific metrics [14,19,20,30]. There were also the ones that focused on academic achievement throughout college [31] or residency program [11,21], by overlooking the number of procedures [32], for example.

In surgery, gender is a substantial subject because the surgical environment is, in general, very masculine. There is still today a discrepancy between women in the early career (residents) and late career (teachers) [33-38], and differences in payment, rates of sexual harassment, rates of sexual abuse, rates of psychological abuse, and so on. The proportion of women in surgery, although it has been increasing in the past few decades [17,20,28,31,32,39]

Motivation is key to good learning, and many learning theories rely on it [40,41]. Without motivation, the learner lacks stimuli and fatally decreases their learning. There is no robust evidence in the literature to support the relationship between learning styles and degree of motivation [16,42-44].

### Limitations

The main limitation of this scoping review is the fact that only English articles were searched, which diminishes the cultural range due to the language barrier, especially if we consider that surgical residency has very different types of models and resources around the world.

### Comparison With Prior Work

No scoping review on learning styles in surgery was ever done.

### Conclusions

This scoping review identified a limited number of studies exploring learning styles in surgery education, with the majority concentrated in the 2010s and predominantly conducted in North America and Europe. The review found that Kolb’s LSI and the VARK instrument were the most frequently used tools, and that research has primarily focused on surgical residents, investigating relationships between learning styles and performance, gender, changes over time, and motivation. The scarcity of studies from other regions, particularly Latin America and Africa, and the limited focus on medical staff and faculty highlight considerable gaps in the current literature. Based on these findings, understanding the characteristics of the existing research landscape is key to informing future studies. Further research is needed to broaden the geographical scope, explore diverse populations within surgical education, and investigate the practical implications of learning style research for enhancing teaching and learning strategies in surgery.

## Supplementary material

10.2196/66766 Multimedia Appendix 1 Detailed search strategy.

10.2196/66766 Multimedia Appendix 2 Search strategy used for one database (PubMed).

10.2196/66766 Checklist 1 PRISMA-ScR checklist.

## Abbreviations

LSI: learning style inventory
MeSH: Medical Subject Headings
PRISMA-P: Preferred Reporting Items for Systematic Reviews and Meta-Analyses Protocols
PRISMA-ScR: Preferred Reporting Items for Systematic Reviews and Meta-Analyses extension for Scoping Reviews
VARK: visual, aural, read, and kinesthetic learning method

## References

[1] Curry L (1983). An organization of learning styles theory and constructs.

[2] Coffield F, Moseley D, Hall E, Ecclestone K (2004). Learning styles and pedagogy in post 16 education: a critical and systematic review.

[3] Pashler H, Mcdaniel M, Rohrer D, Bjork R (2009). Learning styles concepts and evidence. Psychol Sci Public Interest.

[4] Papadatou-Pastou M, Gritzali M, Barrable A (2018). The learning styles educational neuromyth: lack of agreement between teachers’ judgments, self-assessment, and students’ intelligence. Front Educ.

[5] Sternberg RJ, Zhang L (2001). Perspectives on Thinking, Learning, and Cognitive Styles.

[6] Kolb D (1984). Experiential Learning: Experience as the Source of Learning and Development.

[7] Kolb DA (1971). Individual Learning Styles and the Learning Process.

[8] Taylor DCM, Hamdy H (2013). Adult learning theories: implications for learning and teaching in medical education: AMEE Guide No. 83. Med Teach.

[9] Kirschner PA (2017). Stop propagating the learning styles myth. Comput Educ.

[10] Perry WG (1999). Forms of Intellectual and Ethical Development in the College Years: A Scheme.

[11] Reznick RK, MacRae H (2006). Teaching surgical skills — changes in the wind. N Engl J Med.

[12] Frank JR, Jabbour M, Fréchette D, Marks M, Valk N, Bourgeois G (2005). The CanMEDS 2005 Physician Competency Framework Better Standards Better Physicians Better Care Framework.

[13] Jones WHS (1945). The Hippocratic oath - Ludwig Edelstein: the Hippocratic oath. text, translation, and interpretation. pp. vii 64. Baltimore: Johns Hopkins Press, 1943. paper, $1.25. Classical Rev Cambridge University Press. Classical Rev.

[14] Contessa J, Ciardiello KA, Perlman S (2005). Surgery resident learning styles and academic achievement. Curr Surg.

[15] Plovnick MS (1975). Primary care career choices and medical student learning styles. J Med Educ.

[16] Arksey H, O’Malley L (2005). Scoping studies: towards a methodological framework. Int J Soc Res Methodol.

[17] Gouvea Silva G, Costa C da S, Gonçalves BC (2024). Learning styles of medical students, surgical residents, medical staff, and general surgery teachers when learning surgery: protocol for a scoping review. JMIR Res Protoc.

[18] Windsor JA, Diener S, Zoha F (2008). Learning style and laparoscopic experience in psychomotor skill performance using a virtual reality surgical simulator. Am J Surg.

[19] Bell MA, Wales PS, Torbeck LJ, Kunzer JM, Thurston VC, Brokaw JJ (2011). Do personality differences between teachers and learners impact students’ evaluations of a surgery clerkship?. J Surg Educ.

[20] Ahmad HN, Asif M (2018). Medical student’s learning habits: a mixed method study during clinical rotation in general surgery. J Pak Med Assoc.

[21] Bansal R, Mathew KA, Jith A, Narayanan D (2021). A comparison of personality traits, learning style, and perceived stress among surgical and nonsurgical residents in a tertiary care hospital in India. Ind Psychiatry J.

[22] Preece RA, Cope AC (2016). Are surgeons born or made? a comparison of personality traits and learning styles between surgical trainees and medical students. J Surg Educ.

[23] Dankelman J, Chmarra MK, Verdaasdonk EGG, Stassen LPS, Grimbergen CA (2005). Fundamental aspects of learning minimally invasive surgical skills. Minim Invasive Ther Allied Technol.

[24] Martín Parra JI, Toledo Martínez E, Martínez Pérez P (2021). Análisis de los estilos de aprendizaje en un curso de habilidades técnicas laparoscópicas. Implicaciones para el entrenamiento quirúrgico [analysis of learning styles in a laparoscopic technical skills course. Implications for surgical training]. Cirugía Española [Cir Esp].

[25] Pang JHY, Goetz A, Hook L, Joshi ART, Leung PS (2015). Self-awareness of learning styles among surgical trainees. J Am Coll Surg.

[26] Villet R (2020). Teaching surgery in 2020. J Visc Surg.

[27] Metallidou P, Platsidou M (2008). Kolb’s learning style inventory-1985: validity issues and relations with metacognitive knowledge about problem-solving strategies. Learn Individ Differ.

[28] Koob JJ, Funk J (2002). Kolb’s learning style inventory: issues of reliability and validity. Res Soc Work Pract.

[29] Manolis C, Burns DJ, Assudani R, Chinta R (2013). Assessing experiential learning styles: a methodological reconstruction and validation of the Kolb learning style inventory. Learn Individ Differ.

[30] Castillo-Angeles M, Calvillo-Ortiz R, Barrows C, Chaikof EL, Kent TS (2020). The learning environment in surgery clerkship: what are faculty perceptions?. J Surg Educ.

[31] Linn BS, Cohen J, Wirch J, Pratt T, Zeppa R (1979). The relationship of interest in surgery to learning styles, grades and residency choice. Soci Sci Med Part A: Med Psychol Med Sociol.

[32] Quillin RC, Pritts TA, Hanseman DJ, Edwards MJ, Davis BR (2013). How residents learn predicts success in surgical residency. J Surg Educ.

[33] Kim RH, Gilbert T (2015). Learning style preferences of surgical residency applicants. J Surg Res.

[34] Kim RH, Gilbert T, Ristig K (2015). The effect of surgical resident learning style preferences on American board of surgery in-training examination scores. J Surg Educ.

[35] Kim RH, Gilbert T, Ristig K, Chu QD (2013). Surgical resident learning styles: faculty and resident accuracy at identification of preferences and impact on ABSITE scores. J Surg Res.

[36] Mammen JMV, Fischer DR, Anderson A (2007). Learning styles vary among general surgery residents: analysis of 12 years of data. J Surg Educ.

[37] Retrosi G, Morris M, McGavock J (2019). Does personal learning style predict the ability to learn laparoscopic surgery? a pilot study. J Laparoendosc Adv Surg Tech A.

[38] Ballow D, Fang J, Kosarek C, Green T, Tarry W, Tarry S (2015). Mp22-10 impact of matching educational materials to learning style on robotic surgical skills training. J Urol.

[39] Dickinson KJ, Bass BL, Graviss EA, Nguyen DT, Pei KY (2021). How learning preferences and teaching styles influence effectiveness of surgical educators. Am J Surg.

[40] Guariente SMM, Guariente M de M, Moraes A (2020). Perfil sociodemográfico e educacional do estudante ingressante no curso de graduação em medicina de 2004 a 2013: analise documental [socio-demographic profile and educational student newcomer course of graduation in medicine 2004 to 2013: documentary review]. Rev méd Minas Gerais.

[41] Stephens EH, Heisler CA, Temkin SM, Miller P (2020). The current status of women in surgery: how to affect the future. JAMA Surg.

[42] Lim WH, Wong C, Jain SR (2021). The unspoken reality of gender bias in surgery: a qualitative systematic review. PLoS ONE.

[43] Motter SB, Brandão GR, Iaroseski J (2022). Women representation in academic and leadership positions in surgery in Brazil. Am J Surg.

[44] Ferrari L, Mari V, Parini S (2022). Discrimination toward women in surgery: a systematic scoping review. Ann Surg.

