# Supplementary material for: How Learning Styles Characterize Medical Students, Surgical Residents, Medical Staff, and General Surgery Teachers While Learning Surgery: Scoping Review
Source: JMIR Med Educ. 2025 Sep 5;11:e66766. doi: 10.2196/66766 (PMC12413186; doi:10.2196/66766)
Supplement: Multimedia Appendix 1 [file mededu-v11-e66766-s001.docx]

**PUBMED**

Search conducted September 25, 2023.

| Search | query | Records retrieved |
| --- | --- | --- |
| #1 | learn*[Title] | 203,543 |
| #2 | styl*[Title/Abstract] | 74,215 |
| #3 | #1 and #2 | 2,228 |
| #4 | surg*[Title] | 595,939 |
| #5 | #3 and #4 | 57 |
| Final strategy | ("surg*"[Title] AND ("learn*"[Title] AND "english"[Language] AND ("styl*"[Title/Abstract] AND "english"[Language]))) AND (english[Filter]) |  |

**EMBASE**

Search conducted September 25, 2023.

| Search | query | Records retrieved |
| --- | --- | --- |
| #1 | learn*:ti | 243,247 |
| #2 | styl*:ab | 94,340 |
| #3 | #1 and #2 | 2,599 |
| #4 | surg*:ti | 973,654 |
| #5 | #3 and #4 | 67 |
| Final strategy | (‘learn*’:ti and ‘styl*’:ab) and ‘surg*’:ti |  |

**SCOPUS**

Search conducted September 25, 2023.

| Search | query | Records retrieved |
| --- | --- | --- |
| #1 | TITLE ( learn* ) | 984,846 |
| #2 | ABS ( styl* ) | 357,902 |
| #3 | #1 AND #2 | 16,354 |
| #4 | TITLE ( surg* ) | 932,391 |
| #5 | #3 AND #4 | 52 |
| Final strategy | TITLE ( surg* )  AND  ( ABS ( styl* )  AND  TITLE ( learn* ) ) |  |

**WEB OF SCIENCE**

Search conducted September 25, 2023.

| Search | query | Records retrieved |
| --- | --- | --- |
| #1 | TI=(learn*) | 746,534 |
| #2 | AB=(styl*) | 215,792 |
| #3 | #2 AND #1 | 10,166 |
| #4 | TI=(surg*) | 725,525 |
| #5 | #4 AND #3 | 39 |
| Final strategy | ((TI=(surg*)) AND ALL=(AB=(styl*))) AND ALL=(TI=(surg*)) |  |
