# Supplementary material for: How Learning Styles Characterize Medical Students, Surgical Residents, Medical Staff, and General Surgery Teachers While Learning Surgery: Scoping Review
Source: JMIR Med Educ. 2025 Sep 5;11:e66766. doi: 10.2196/66766 (PMC12413186; doi:10.2196/66766)
Supplement: Multimedia Appendix 2 [file mededu-v11-e66766-s002.docx]

**Search strategy for PUBMED**

Search conducted September 25, 2023.

| Search | query | Records retrieved |
| --- | --- | --- |
| #1 | learn*[Title] | 203,543 |
| #2 | styl*[Title/Abstract] | 74,215 |
| #3 | #1 and #2 | 2,228 |
| #4 | surg*[Title] | 595,939 |
| #5 | #3 and #4 | 57 |
| Final strategy | ("surg*"[Title] AND ("learn*"[Title] AND "english"[Language] AND ("styl*"[Title/Abstract] AND "english"[Language]))) AND (english[Filter]) |  |
